# UHPLC-QTOF-MS-Based Targeted Metabolomics Provides Novel Insights into the Accumulative Mechanism of Soil Types on the Bioactive Components of *Salvia miltiorrhiza*

**DOI:** 10.3390/molecules29174016

**Published:** 2024-08-25

**Authors:** Mengmeng Hou, Dan Gao, Weixu Chen, Wenjun Jiang, Dade Yu, Xiwen Li

**Affiliations:** 1 Institute of Chinese Materia Medica, China Academy of Chinese Medical Sciences, Beijing 100700, China; hmm199916@163.com (M.H.); dgao@icmm.ac.cn (D.G.); wenjunjiang0927@gmail.com (W.J.); 2 College of Traditional Chinese Medicine, Henan University of Chinese Medicine, Zhengzhou 450046, China; 3 China Shangyao Huayu (Linyi) Traditional Chinese Medicine Resources Co., Ltd., Linyi 273300, China; chwxu2006chwxu@126.com

**Keywords:** HPLC, soil and plant ecological processes, active plant constituents, soil fertility, heavy metals

## Abstract

The root of *Salvia miltiorrhiza* Bunge (SMB) has been widely used to treat cardiovascular diseases. However, the contents of secondary metabolites in the roots from different production areas are significantly different, and the impact of soil factors on this accumulation remains unclear. Therefore, this study aimed to elucidate the regularity of variation between the active components and soil factors through targeted metabolomics and chemical dosimetry. Soils were collected from five different cities (A, B, C, D, and E) and transplanted into the study area. The results showed that there were significant differences in the soil fertility characteristics and heavy metal pollution levels in different soils. Ten water- and twelve lipid-soluble metabolites were identified in SMBs grown in all soil types. SMBs from D cities exhibited the highest total tanshinone content (*p* < 0.05). The salvianolic acid B content in SMBs from E cities was the highest (*p* < 0.05). Interestingly, correlation analysis revealed a significant negative correlation between the accumulation of lipid-soluble and water-soluble metabolites. Double-matrix correlation analysis demonstrated that available potassium (AK) was significantly negatively correlated with salvianolic acid B (r = −0.80, *p* = 0.0004) and positively correlated with tanshinone IIA (r = 0.66, *p* = 0.008). Conversely, cadmium (Cd) and cuprum (Cu) were significantly positively and negatively correlated with salvianolic acid B (r = 0.96, *p* < 0.0001 and r = 0.72, *p* = 0.0024) and tanshinone IIA (r = 0.40, *p* = 0.14 and r = 0.73, *p* = 0.0018), respectively. Mantel’s test indicated that AK (r > 0.52, *p* < 0.001), Cu (r > 0.60, *p* < 0.005), and Cd (r > 0.31, *p* < 0.05) were the primary drivers of the differences in the active components of SMBs. These findings provide a theoretical framework for modulating targeted metabolites of SMB through soil factors, with significant implications for the cultivation and quality control of medicinal plants.

## 1. Introduction

*Salvia miltiorrhiza* Bunge (SMB) is a plant widely used in traditional Chinese medicine for the treatment of cardiovascular diseases (CVD) [1,2]. Due to its therapuetic benefits, it has been incorporated into many pharmacopeias worldwide [3], with its popularity ever increasing due to the constant rise in morbidity and mortality caused by CVD [2,4]. Its effectiveness can be attributed to its water-soluble phenolic acids and lipid-soluble ketones, which have unique effects on various diseases. The water-soluble compounds include salvianolic acid, caffeic acid, and salvianolic acid B. Recent studies have indicated that these components have anti-arrhythmic properties, thereby dilating coronary arteries [5,6,7], regulating blood lipids, improving microcirculation, reducing blood pressure, and inhibiting platelet aggregation. Moreover, water solubility has a positive effect on chest pain, coronary heart disease, and angina pectoris caused by blood stasis. The lipid-soluble compounds present in SMB are mainly pheromones, such as tanshinone I, tanshinone IIA, and cryptotanshinone. Unlike water-soluble compounds, lipid-soluble compounds usually have more direct biological activities, and they can prevent and treat CVDs by acting on multiple biological pathways, such as anti-inflammatory, antioxidant, and antitumor [6,8,9]. Simultaneously, the proportion of the active ingredients in SMB is critical for determining its clinical efficacy [10]. 

The natural distribution of SMBs ranges from the east of the Taihang Mountains to the west of the central Qinling Mountains in China. Shandong Province is located in an optimal growth area, where SMBs are intensively cultivated. Farmland cultivation is the primary method for producing SMB to meet the yield demands [11]. However, intensive farming practices can deplete soil nutrients [12,13], as plants selectively absorb elements, leading to a decline in soil element diversity and content in high-production areas. This, in turn, contributes to the compositional variations among different cultivation regions [14,15]. Simultaneously, heavy metals such as Cuprum (Cu) and Cadmium (Cd) in the soil can also affect the content of active components in the roots of SMB [16,17,18]. Therefore, it is necessary to investigate the relationship between soil factors and the active components of SMB.

The active components of plants are affected by multi-dimensional interactions such as soil factors, species genetics, habitat, climate, and habitat management [19,20,21]. Liu [22] indicated that rosmarinic acid and salvianolic acid B were positively correlated with latitude, whereas tanshinone was negatively correlated with annual relative humidity. Zhang [23] observed that under mild drought conditions, the phenolic acid components of SMB were significantly upregulated during biosynthesis. Zhao [24] determined different SMB genotypes and locations influenced the content of medicinal ingredients found within the plant. This indicates that different soil factors have different effects on the accumulation of SMB components. He et al. [25] indicated that tanshinone IIA was significantly and positively correlated with soil pH, soil organic matter (SOM), available nitrogen fertilizer (AN), and aluminum (Al), whereas rosmarinic acid showed the opposite responses to SOM and Al. Liu and Zhang [22] demonstrated that tanshinone is positively correlated with soil available magnesium (Mg) and negatively correlated with soil cadmium and total available nitrogen (TAN) [26]. In contrast, the reaction of phenolic acid with TAN and Mg was the opposite of that of tanshinone. As indicated, the active components of SMBs are affected by genotype, climate, field management, and other factors [21,22,23,24,25,26,27]. Previous studies on the correlations between soil factors and active components have not excluded these complex effects.

To address these constraints, SMB materials of the same genotype will be collected and these will be exposed to controlled and consistent climatic factors. Moreover, uniform field management will be maintained to elucidate the response mechanisms of SMB secondary metabolites to soil factors. This study aims to achieve the following: (1) investigate the accumulation patterns of SMB chemical components under different soil conditions at the same cultivation site using ultra-high performance liquid chromatography–quadrupole time-of-flight mass spectrometry (UHPLC-QTOF-MS); (2) profile the response patterns of soil factors to the biosynthesis and accumulation of SMB active components through correlation analysis and chemometrics; and (3) scientifically guide field management by exploring the key soil drivers that affect secondary metabolites.

## 2. Results

### 2.1. Comparative Analysis of Different Soil Factors from the Five Soils

**Figure 1:**
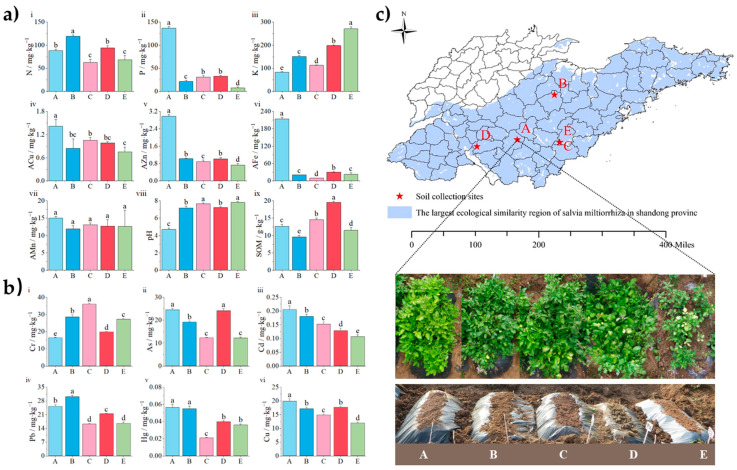
Samples collected from five sites (A, B, C, D, and E) in Shandong Province. (**a**) Physical and chemical properties of the soils. AN: alkaline nitrogen; AP: available phosphorus; AK: available potassium; ACu: available copper; AZn: available zinc; AFe: available iron; AMn: available manganese; SOM: soil organic matter. (**b**) Contents of soil heavy metals among different soil samples. Cd: Cadmium; Cr: Chromium; As: Arsenic; Pb: Lead; Hg: Hydrargyrum; Cu: Cuprum. Significant differences between soils were indicated by the least significant difference (LSD) test, with different lowercase letters indicating *p* < 0.05. (**c**) The five locations where soils were collected and the planting point in Site A where all soils were transported to.

The pH, organic matter, heavy metals, and pollutant contents in the different soil samples are shown in Figure 1a,b. The amount of AN (*p* < 0.05, N = 119.3 mg·kg^−1^), available phosphorus (AP; *p* < 0.05, P = 136.6 mg·kg^−1^), and AK (*p* < 0.05, K = 270.3 mg·kg^−1^) in soils B, A, and E was significantly higher than that of the other soils, respectively. The N:P:K content of soils A, B, C, D, and E were 1.1:1.6:1, 5.6:1:7.0, 2:1:3.6, 2.9:1:6.0, and 9.2:1:36.2, respectively. The ratio of N and P in soils A, C, and D was maintained between 1.1 and 6.0 times, while the ratio of soil E was significantly different (9.2–36.2 times). The proportion of the difference between soil B was between these two values. Except for available manganese (AMn), there were significant differences in the other available elements [available copper (ACu), available iron (AFe), available zinc (AZn), AP, and AK] in the different soils (*p* < 0.05). The contents of ACu, AZn, and AFe in soil A were significantly higher than those in the other four soils (*p* < 0.05, ACu = 1.4 mg·kg^−1^, AZn = 3.0 mg·kg^−1^, AFe = 3.0 mg·kg^−1^). Simultaneously, there were certain differences between the pH of different places, except soil A, which was acidic (pH = 4.71); the rest of the soils were weakly alkaline (pH = 7.17~7.80). Figure 1a shows that soil D had the highest SOM (*p* < 0.05, 19.57) content, whereas soil B (*p* < 0.05, 9.61) had the lowest SOM content. Heavy metals and pollutants were analyzed, and the results are shown in Figure 1b. Soil A had the highest As, Cd, Hg, and Cu contents, whereas lead (Pb) and hydrargyrum (Hg) were highest in soil B. Soils C and E had the highest Cr, whereas soil D had the highest Ar content. Notably, the heavy metals in the five soils were all less than the screening value of the soil pollution risk for agricultural land in the GB 15618-2018 soil environmental quality. Soil pollution risk control standard for agricultural land (trial), indicating that the soil pollution risk of the five producing areas is low and can be used as farmland soil.

### 2.2. Changes in Salvianolic Acid B and Tanshinone IIA in Different Soils

The SMB components from different soils can be divided into three main categories. The first category includes high salvianolic acid B and low tanshinone IIA levels. From the results, salvianolic acid B content was higher in cultivated soil E compared to that of soils A, C, and D (*p* < 0.05) (Table 1). Additionally, the total tanshinone content was highest in cultivated soils B and E (*p* < 0.05). However, the total tanshinone content in SMB-cultivated soils A, C, and D was at least 0.25 times higher than that of soils B and E. The second category includes low salvianolic acid B and tanshinone IIA. In this category, cultivated soil B fitted into this category as it demonstrated low concentrations for both contents. The third category included low salvianolic acid B and high tanshinone IIA contents. The salvianolic acid B content in cultivated soil E was the highest (*p* < 0.05), whereas the total tanshinone content was the lowest (*p* < 0.05). The total tanshinone content in cultivated soil E was at least 0.25 times higher than that of the SMBs from the other two soils. The total tanshinone content in cultivated soil E was only 59.2% of that in soil D. 

The SMBs cultivated in the five soils exceeded 6% for salvianolic acid B, which was significantly higher than the lower limits (3%) and the total tanshinone content exceeded 0.6%, which was lower than the total tanshinone content (0.25%) stipulated in the Chinese Pharmacopeia (2020 Edition) [28]. Further analysis showed that the ratio of salvianolic acid B to total tanshinone cultivated in soil A was the lowest (7.03); B, C, and D were in the medium range of 8.87~9.34; and soil E was the highest (15.99). These results show that the environmental conditions of soil E were conducive to the accumulation of salvianolic acid B in SMBs, whereas soils A, C, and D tended to promote the accumulation of tanshinone IIA in SMBs. Soil B showed poor enrichment of both components in the SMB. 

**Figure 2:**
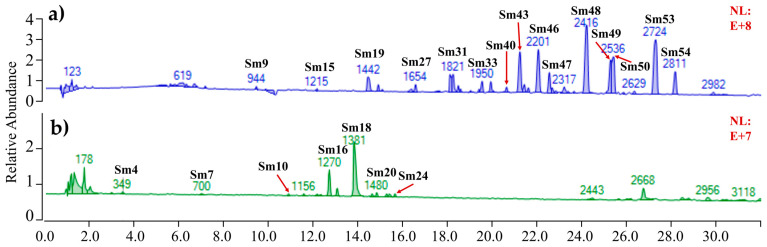
Total Ion Chromatography (TIC) chromatogram in positive mode (blue colour/ **a**) and negative mode (green colour/ **b**) of SMB. The peak numbers are based on Table 2.

Based on the HPLC analysis, 56 common peaks were isolated from 15 SMB samples and were labeled as Sm1–Sm56 according to the retention time (Table S1; Figures S1 and S2). SMB samples were identified using UHPLC-QTOF-MS. The deprotonated or protonated molecules [M + H]^+^ and [M + Na]^+^ [24,29] were selected as the precursor ions for collision-induced dissociation (CID) fragmentation to generate the mass spectrum/mass spectrum product ion spectrum. The decomposition pathway was analyzed using rosmarinic acid and cryptotanshinone. The [M − H] ion loss of the water-soluble rosmarinic acid at *m*/*z* 359 corresponds to the [M − H − 198] ion generation (*m*/*z* 161) and (*m*/*z* 107) fragment ions of Danshensu (198 Da) [30] (Table 2). The [M+H] ions of lipid-soluble cryptotanshinone at *m*/*z* 297 are generated [M − H − H_2_O] (*m*/*z* 279) and M − H − H_2_O − CO] (*m*/*z* 251) due to the presence of hydroxyl and ether bonds [30] (Table 2). The most prominent product ions were selected for MSn analysis. By comparison with reference to the standards of salvianolic acid B, tanshinone IIA, and other relevant literature data [24,29,30,31,32,33,34,35], 22 compounds were identified, including 10 phenolic acids and 12 diterpenoid quinones (Figure 2, Table 2). The phenolic acid components included danshensu (Sm4), caffeic acid (Sm7), salvianolic acid F (Sm9), hydrosalvianolic acid B (Sm10), rosmarinic acid (Sm15), lithospermic acid (Sm16), salvianolic acid B (Sm18), salvianolic acid E (Sm19), isosalvianolic acid B (Sm20), and methyl salvianolic acid I/H (Sm24). Diterpene quinone compounds containing tanshinone VI (Sm27), tanshinone IIB (Sm31), methyl dihydronortanshinonate (Sm33), salvianolic aldehyde (Sm40), 15th and 16th—dihydrotanshinone I (Sm43), trijuganone B (Sm46) and methyl tanshinonate (Sm47), crypto-tanshinone (Sm48), 1, 2-dihydrotanshinone I (Sm49), 3,4-dihydrotanshinone I (Sm50), tanshinone IIA (Sm53), and miltirone (Sm54) (Table S2). 

### 2.3. The Biosynthetic Pathways of Salvianolic Acid B and Tanshinone I of SMB Planted in Different Soils Were Different

**Figure 3:**
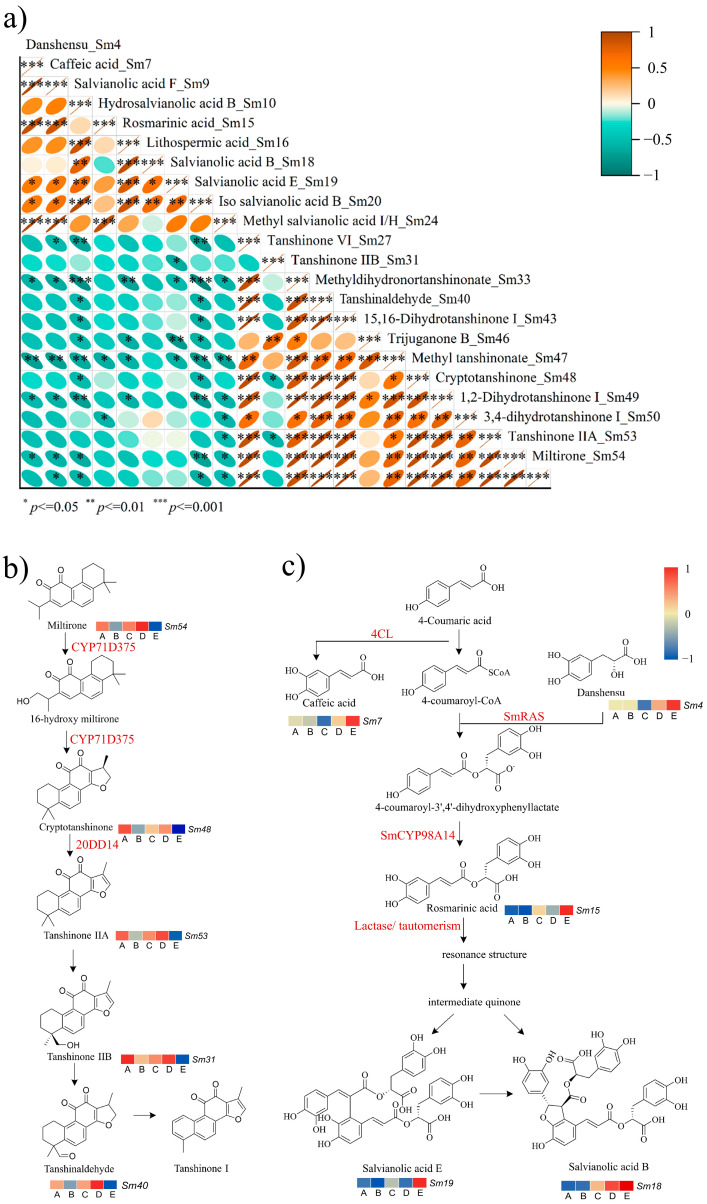
Correlation analysis of active components identified in SMBs. (**a**) The correlation plots show Pearson correlation values for the 22 bioactive components of SMB. The color and size of the oval chart denote the magnitude and direction of the relationship. (**b**) The biosynthetic pathways of tanshinone I. (**c**) The biosynthetic pathways of salvianolic acid. The Pearson correlation coefficient measures the degree of correlation between two variables. A one-way analysis of variance (one-way ANOVA) was used for statistical analysis. * *p* < 0.05, ** *p* < 0.01, *** *p* < 0.001 were considered statistically significant.

The Pearson correlation coefficient (r) was calculated to determine whether the linear relationship between the two variables was strong or weak and whether the linear strength was statistically significant. Twenty-two identified active ingredients were compared in a pairwise manner to determine the correlation between ingredient levels and their significance. Significant positive correlations were determined among most of the water-soluble compounds in the top eight phenolic acids. Similarly, significant positive correlations were observed among the topmost diterpenoid lipid-soluble compounds from the final peak (Figure 3a). Interestingly, correlation analysis revealed a significant negative correlation between the accumulation of lipid- and water-soluble metabolites. In particular, methyl salvianolic acid I/H (Sm24) was positively correlated with most lipid-soluble components (*p* < 0.05). Based on the SMB tanshinone I biosynthesis pathway, SMBs cultivated in soils A, C, and D had higher lipid-soluble components than compared to those cultivated in soil B, which had medium lipid-soluble components, and those cultivated in soil E had the lowest lipid-soluble components. The content of SMBs cultivated in soils A and B showed an upward trend from the initial end (miltirone) to the synthetic end (tanshinone IIB), whereas the accumulation of lipid-soluble fractions of SMBs cultivated in soils C and D first decreased and then increased (Figure 3b). Based on the water-soluble biosynthesis pathway of SMB, the content of water-soluble components of SMBs cultivated in soils A and B was the first to gradually decrease. However, the accumulation of SMBs in soil C increased from low to medium. The active component content of SMBs cultivated in soil D was polarized from rosmarinic acid, in which the accumulation of salvianolic acid B showed an upward trend and that of salvianolic acid E showed a downward trend (Figure 3c). According to the comprehensive analysis in Figure 3b,c, the relative contents of SMB water- and lipid-soluble components from the five soils showed an opposite trend, which is similar to the result in Figure 3a. Most of the SMB lipid- and water-soluble components showed significant negative correlations (*p* < 0.05, −1 < r < −0.6). In conclusion, based on the composition and proportion of different soils, the synthetic accumulation of SMB lipid- and water-soluble components may show decreasing and increasing trends, respectively. Such changes can be attributed to the combined effects of nutrients, trace elements, organic matter, and heavy metal pollutants in the soil.

### 2.4. Chemometrics Analysis

**Figure 4:**
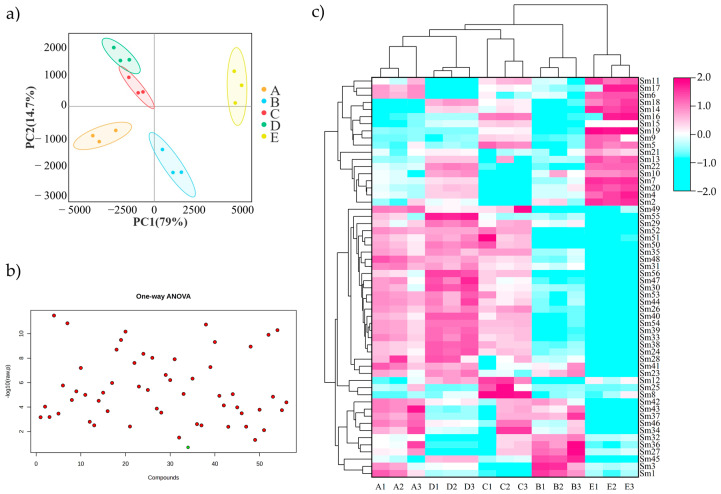
Clustering of active components of Salvia divinorum in five soils. (**a**) Principal component analysis of active components in samples from five production areas. (**b**) Fifty-six common components one-way ANOVA. The 56 points represent the 56 active components of SMB. Red indicates significant differences between the five soils, and green indicates no significant differences (*p* < 0.05). (**c**) Cluster analysis of active components in five production areas. A1–E3 represents soil collected from five different cities (A, B, C, D, and E). Sm1-Sm56 are sorted according to the order of peaks, of which Sm24 is methyl salvianolic acid I/H and Sm27 is tanshinone VI. Based on the analysis of chemical analysis of SMB, Sm24 and its before are water-soluble components, and Sm27 and its after are lipid-soluble diterpene quinone components.

Similar correlation coefficients were calculated based on 56 shared chemical components (Table S1). The results showed that the measured values of the 15 samples were in the range of 0.949–0.999, and the relative standard deviation (RSD) of the total peak area for each chromatographic fingerprint was only 0.02%. The chemical types of the common active ingredients in the 15 SMB samples were very similar, but the specific amounts of the active ingredients were quite different. To gain a deeper understanding of this discrepancy, a principal component analysis (PCA) was performed based on the 56 common peak areas of the 15 SMB samples. PCA showed that all SMBs cultivated in the same soil clustered into one group, and there was an obvious separation among the five soils. On the PC1 axis, the components of SMBs cultivated in soil E and those cultivated in the other four soils were negatively distributed (Figure 4a).

Based on the one-way ANOVA, it was determined that 55 of the 56 chemical components of SMBs cultivated in the five soils were significantly different (*p* < 0.05; Figure 4b). Therefore, a cluster heatmap analysis was performed based on the 56 shared chemical components. Three duplicate samples of SMBs cultivated in each soil type were clustered horizontally into a small branch (Figure 4c), showing the uniformity of SMBs growing in a specific soil type. The SMBs samples cultivated in soils E, B, and A; D; and C clustered into Classes I, II, and III, respectively. This is consistent with the PCA results. On the PC1 axis, the SMB components in cultivated soil E and in the other four soils showed a negative distribution (Figure 4a). On the PC2 axis, the SMB components in soil B and the other four soils were negatively distributed (Figure 4a). The 18 chemical components before Sm24 (methyl salvianolic acid I/H) clustered into one group (Figure 4c) and were mostly water-soluble compounds. The remaining 38 chemical components clustered into another group (Figure 4c), which were mostly lipid-soluble components with a small portion of water-soluble components. In Class II, 21 chemical components after Sm27 (tanshinone VI) and Sm23, Sm24, and Sm26 clustered into one branch, and nine chemical components after Sm27 (tanshinone VI), Sm1, and Sm3 clustered into a second branch. The overall analysis showed that SMBs cultivated in soil E were separated into Class I and Class II, with a high content of Class I water-soluble components and a low content of Class II lipid-soluble components (Figure 4c). SMBs cultivated in soil B had a low water-soluble component content in vertical Class I and a low content of lipid-soluble components in the first branch of vertical Class II, indicating that the content levels of most lipid- and water-soluble components of SMBs cultivated in soil B were low. SMBs cultivated in soils A, D, and C showed a relatively low content of water-soluble components in Class I and a high content of lipid-soluble components in the first branch of Class II. The SMB samples cultivated in soil A had higher content levels than those cultivated in soils D and C in the second branch of Class II. The results showed that the composition of SMB samples could be affected by different soils, and the water- and lipid-soluble components showed significant differences in the samples cultivated in different soils.

### 2.5. Correlation Analysis of SMB Components and Soil Factors

**Figure 5:**
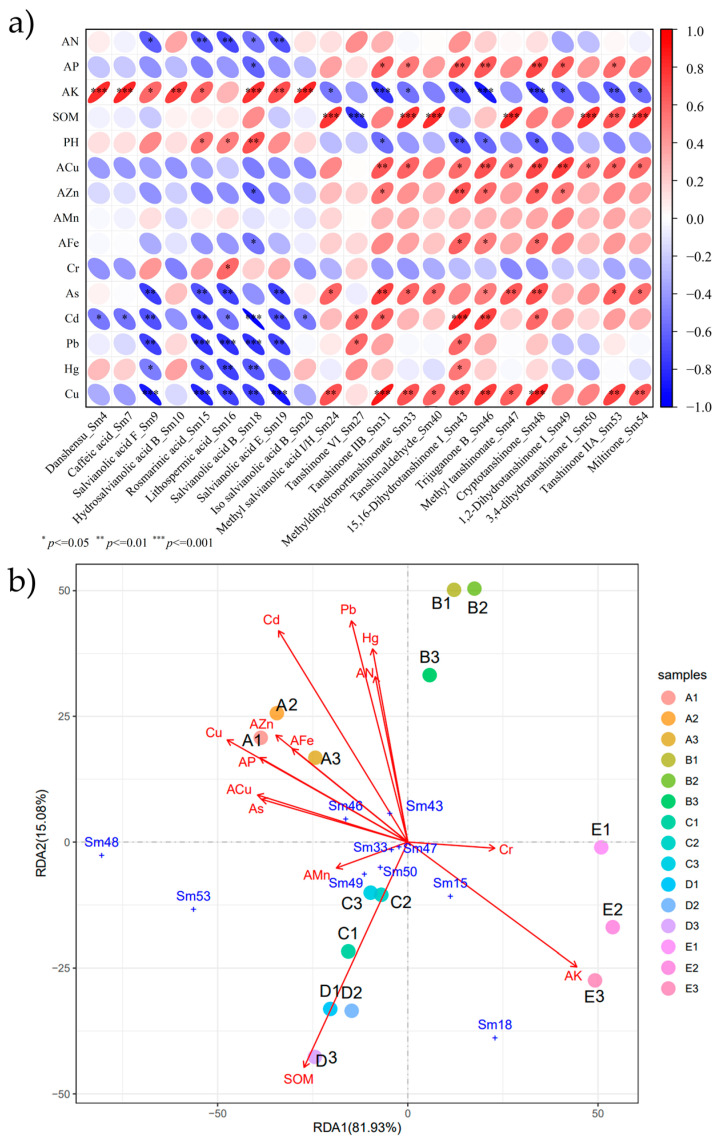
Correlations of bioactive compounds and soil properties. (**a**) Heat map of correlation of 22 active ingredients with soil factors. Different ellipse sizes represent the degree of correlation. (**b**) Inferred RDA plot. The red arrows indicate the soil factors; their length corresponds to the correlation between the soil factors and the content of the 22 active ingredients in Salvia divinorum; and the angle between the arrows and the axes reflects the correlation between them. Nine sample locations are indicated by colored circles.

Pearson’s test was used to examine the correlation between 15 soil properties and 22 chemical components. Except for AN, Cr, and AMn, all other elements showed significant correlations with the 22 components. Among the macroelements, AK was most correlated with the index component (r^2^ = 0.823, *p* < 0.001). The SOM and index components also showed significant correlations (r^2^ = 0.877, *p* < 0.001) (Figure 5a). Among the heavy metals, Cd (r^2^ = 0.933, *p* < 0.001) and Cu (r^2^ = 0.852, *p* < 0.001) had the greatest impact on composition. However, based on the overall observation of the correlation, it was determined that the water- and lipid-soluble fractions showed different trends in response to soil factors. AK was positively correlated with water-soluble components, whereas AN and the heavy metals As, Cd, Pb, Hg, and Cu were negatively correlated (Figure 5a). In contrast, AK was negatively correlated with most lipid-soluble components, and AP and heavy metals As, Cd, and Cu were positively correlated with most lipid-soluble components. In addition, there was a significant negative correlation between the trace elements ACu, AZn, and AFe and small lipid-soluble components. 

Redundancy analysis (RDA) and canonical correspondence analysis (CCA) are multivariate statistical methods commonly used to analyze principal components and linear relationships between multiple variable sets. In this study, species-sample data were used for detrended correspondence analysis (DCA), and the size of the first axis of the lengths of gradient was 0.99 (Table S3). Therefore, RDA was used to explore the relationship between the SMB metabolite set, soil cause subset, and SMB source set [36]. The RDA results showed that the total variance of RDA1 and RDA2 was 97.01% (Figure 5b). There were no significant differences between AMn, Cr, and most of the components, which is consistent with the results shown in Figure 5a. These correlation analyses suggest that AN fertilizers have minor effects on the biosynthesis of SMB components and that AP and AK fertilizers may lead to differences in the biosynthesis of SMB components. SOM and trace elements slightly increased the accumulation of lipid-soluble components. However, the accumulation of water-soluble components was not significant. The accumulation of heavy metals decreased the content of water-soluble components and increased lipid-soluble components. 

### 2.6. Soil Drivers of Water- and Lipid-Soluble Constituents of SMBs

**Figure 6:**
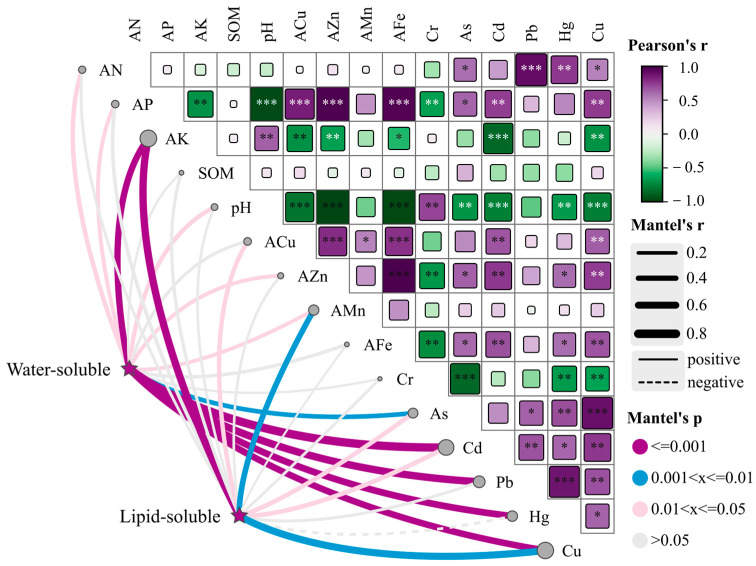
Soil drivers of lipid-soluble and water-soluble components of SMBs. AN: alkaline nitrogen; AP: available phosphorus; AK: available potassium; ACu: available copper; AZn: available zinc; AFe: available iron; AMn: available manganese; Cd: Cadmium; Cr: Chromium; As: Arsenic; Pb: Lead; Hg: Hydrargyrum; Cu: Cuprum. A two-by-two comparison of soil factors is shown, with color gradients indicating Spearman correlation coefficients. Categorical (based on heat maps of active ingredient correlations and cluster analysis across sites) composition was correlated with each soil factor by a partial (geographic distance corrected) Mantel’s test. Edge width corresponds to Mantel’s r statistic corresponding to the distance correlation, and edge color indicates statistical significance based on 9999 alignments. Solid lines represent having a positive correlation and dashed lines represent having an inverse correlation. A one-way analysis of variance (one-way ANOVA) was used for statistical analysis. * *p* < 0.05, ** *p* < 0.01, *** *p* < 0.001 were considered statistically significant.

Mantel’s test was used to verify the correlation between two matrices. In ecology, it is often used to test the correlation between the community and environmental variable distance matrix. The larger the correlation coefficient (r) of the Mantel’s test and the smaller the *p* value, the greater the influence of environmental factors on microbial communities. Partial analysis using Mantel’s test can eliminate the interference of autocorrelation between environmental factors. In this study, Mantel’s test was used to examine the correlation between the distance matrix of SMB metabolites and soil factor variables. The correlation analysis of soil factors showed that, excluding Cr, heavy metals were positively correlated with each other, and As, Hg, and Cu were negatively correlated with Cr (*p* < 0.01) (Figure 6). Except for Cr, heavy metals were positively correlated with AN, AP, ACu, and AZn and negatively correlated with AK and pH. There were differences in environmental factors affecting the biosynthesis and accumulation of water-soluble components of SMB and lipid-soluble components. Mantel test analysis showed that for water-soluble heavy metals, Cd was the most important driving factor affecting biosynthesis and accumulation (*p* < 0.001, r = 0.77). This was followed by Pb (*p* < 0.001, r = 0.66), Hg (*p* < 0.001, r = 0.57), Cu (*p* < 0.001, r = 0.55), and AK (*p* < 0.001, r = 0.53). Among the lipid-soluble components, AK was the most important factor affecting biosynthesis and accumulation (*p* < 0.001; r = 0.65). It was followed by Cu (*p* = 0.002, r = 0.61), AMn (*p* = 0.004, r = 0.43), Cd (*p* = 0.018, r = 0.32), and As (*p* = 0.013, r = 0.31). Soil environmental factors (AK, Cu, and Cd) were the main driving factors affecting the biosynthesis and accumulation of water- and lipid-soluble components of SMB (Figure 6). 

## 3. Discussion

SMB is the gold standard for treating CVDs [37]. Rhizomes are rich in water- and lipid-soluble active tanshinones [37,38]. Studies have shown that phenolic acids and tanshinones are the main bioactive ingredients used to improve blood circulation, treat stasis removal [39], and enhance cardiovascular protection [40,41]. Simultaneously, chemical composition is clearly required for content determination in the Chinese National Pharmacopeia [28]. He and Liang [25,42] indicated a high salvianolic acid B and low tanshinone IIA content in cultivated and wild SMBs in Shandong Province, the main production area in China. Additionally, there was a large variation range in tanshinone content in wild SMB. In this study, it was observed that the active components of SMBs from different soils in the Shandong Province were very different at the same time and place, with the same varieties and management methods. Among them, the total ketones of SMB had a larger variation range than compared to that of phenolic acids, which is similar to previous studies [25,42]. Zhang [26] reported that, among 41 wild SMB samples collected from China, the salvianolic acid B was concentrated between 1.39% and 11.10%, with an average value of 6.31. In this study, the salvianolic acid B from five different soils ranged from 7.1 to 9.7%, and the SMBs ketone content ranged from 0.61 to 1.00%. The content of the active ingredients met the requirements of the Chinese Pharmacopeia (2020 edition). The proportion of SMBs cultivated in soil E was far beyond this range. The salvianolic acid B content of SMB was highest and lowest in cultivated soil E, compared to that of the other soils. These results indicate that, compared with lipid-soluble components, soil E was more inclined to synthesize and accumulate water-soluble components in SMB. Notably, SMBs cultivated in soil D demonstrated a 94.42% increase in tanshinone content compared to those grown in soil E; this was an overall increase of 68.85%. Furthermore, except for Cr, it was determined that other heavy metal pollutants in soil D were significantly higher than those in soil E; therefore, this indicates that this is potentially one of the factors causing this substantial increase. 

In the present study, a significant positive correlation was observed between the water solubilities of the different phenolic acids. There was also a significant positive correlation between the lipid solubilities of the diterpenoid quinones. This finding is similar to that of previous studies. Zhou and Deng [43,44] showed that most water-soluble phenolic acids in SMB were synthesized by modification of the precursor compound, romatinic acid. Research on the synthesis pathways of lipid-soluble components is relatively scarce. However, Ma and Zhang [45,46] indicated that most lipid-soluble components of SMB had a common mother nucleus. One of the few exceptions is compound Sm24 methyl salvianolic acid I/H, as studies have shown that salvianolic acid I can be used to inhibit cervical intraepithelial neoplastic cells [47]. Methyl salvianolic acid (I/H) is a water-soluble phenolic acid in SMB that is positively correlated with most lipid-soluble compounds. Studies have shown that salvianolic acid I uses ferulic acid as a precursor for synthesis [48], which may be one of the factors leading to the differences between salvianolic acid and other water-soluble compounds.

The physical and chemical properties, organic matter content, and inorganic elements of the soil can have a significant impact on the active ingredients of Chinese herbal medicines [14,49]. In this study, the content of compound components in SMB samples from the same germplasm and place of origin was significantly different, indicating that soil has an impact on the content of the effective components of SMB under the same climatic conditions. Salvianolic acid B content in the roots of SMB was positively correlated with AK and soil pH (*p* < 0.05) and negatively correlated with AN, AK, AZn, and AFe in the soil (*p* < 0.05). The tanshinone IIA and cryptotanshinone contents were positively correlated with SOM and ACu in the soil (*p* < 0.05) and negatively correlated with AK in the soil (*p* < 0.05). It should be noted that most of the elements are mutually exclusive for the biosynthesis of water- and lipid-soluble components. 

Heavy metals and pollutants can enter soil through atmospheric sedimentation [50], water source discharge [51], fertilizers, and pesticides [52]. It directly affects plant physiology and growth [53,54,55]. These pollutants also stimulate the selective secretion of secondary metabolites by medicinal plants [17,55,56]. In this study, water solubility showed a significant negative correlation with As, Cd, Pb, Hg, and Cu. Surprisingly, the lipid-soluble fraction exhibited the opposite trend (Figure 5). Among them, the effects of As, Cd, and Cu on the chemical constituents of SMB were the most significant. In this study, Cu reduced the biosynthesis and accumulation of salvianolic acid F, rosmarinic acid, lithospermic acid, salvianolic acid B, and salvianolic acid E in the SMB. In contrast, salvianolic acid I/H, tanshinone IIB, methyldihydronortanshinonate, tanshinaldehyde, 15-dihydrotanshinone I, trijuganone B, methyl tanshinonate, cryptotanshinone, tanshinone type IIA, and miltirone were biosynthesized and accumulated. LIU [22] determined that tanshinone content was positively correlated with Cu, and REN [16] indicated that Cu increased tanshinone II and total tanshinone contents but had no significant effect on salvianolic acid B content. The results of this study are in good agreement with those of previous studies: Cu content is negatively correlated with the biosynthesis and accumulation of water-soluble components in the SMB and positively correlated with the biosynthesis and accumulation of lipid-soluble components in the SMB. In addition to reducing the effects of salvianolic acid B and 15,16-dihydrotanshinone I, As showed a consistent trend compared with Cd in the other compounds mentioned above. At present, there is no literature on As pollution in the SMB, but Bhardwaj [57] observed that the flavonol concentration in radishes decreased sharply under arsenic stress. Lu [58] indicated that As stress increased the accumulation of eight terpenoids in the roots, indicating that plants would increase or decrease the secretion of secondary metabolites in response to As stress. Compared to the effect of Cu on the secondary metabolites of SMB, Cd was negatively correlated with danshensu, caffeic acid, isosalvianolic acid B, and tanshinone VI. Chai [56] showed that, compared with the control, the application of Cd increased the content of SMB cryptotanshinone and tanshinone IIA but decreased the content of salvianolic acid B. Yuan [56] demonstrated that the ketone content in SMB first decreased and then increased with the increase in Cd, and our results were in good agreement with previous studies.

These correlations indicate that the environmental parameters in different soils change the accumulation of lipid- and water-soluble components in the roots of SMB, especially the accumulation of the heavy metal Cd, trace element AMn, and nutrient element AK. High AK concentrations in the soil promoted an increase in salvianolic acid B content but suppressed the accumulation of tanshinone IIA and cryptotanshinone. Neutral soil is generally believed to be more suitable for plant growth, and medicinal plants have different effects on the chemical composition. Su et al. [14] observed that acidic soil is conducive to the accumulation of volatile oil components of ‘Chachi’, but some studies have shown that SMB is suitable for growth in neutral soil. In this study, soil A belongs to the weak acid soil, and SMBs cultivated in soil A have low salvianolic acid composition, which results in a difference between the results of ‘Chachi’. The use of fruits and rhizomes may be one of the factors leading to differences in the results. In terms of soil heavy metals and pollutants, the Dan phenolic acid content of B and the contents of Cd, Pb, Hg, and Cu showed significant negative correlations (*p* < 0.05), and tanshinone, tanshinone, As, and Cu showed significant positive correlations. When water- and lipid-soluble components as a whole were investigated as a whole, it was determined that for water-soluble components, the variation in AK content in soil increased the coefficient of variation of water- and lipid-soluble components.

## 4. Materials and Methods

### 4.1. Study Sites

The cultivation site is located in central Shandong Province (East China), which has a warm temperate monsoon climate. The following five sample areas were selected for soil collection: Pingyi County (soil A; E 35.498 N 117.777), Linqu County (soil B; E 36.522 N 118.627), Zoucheng City (soil D; E 35.343 N 116.86), and Ju County 1 (soil C; E 35.437 N 118.746) and Ju County 2 (soil E; 35.438 N 118.743) of Shandong Province (Figure 1c). These specific areas were selected because they are deemed ecologically suitable for SMB farming and cultivation. Considering that a small range of soil differences still exists in the same production area, two soil samples were selected from Ju County.

### 4.2. Soil Collection and Experimental Set-Up

In March 2022, soil samples were collected from 8 to 10 representative plots. At each sampling point, the five-point sampling method was used to collect the surface soil (1–30 cm). Surface weeds were removed. Non-soil components such as plant residues were selected, and the remaining soil was used as native subsoil. All soil samples from the five locations were transported to the study site in location A (Figure 1c). As shown in Figure 1c, a pit (length × width × height = 8 m × 5 m × 1.5 m) was dug and filled with soil from the five locations. Each soil sample was filled to a ridge, with a length of 4 m, a width of 0.85 m at the bottom, and a width of 0.50 m at the top. The height of each ridge was 2 m based on the average length and width of the SMB roots. Black light–opaque plastic films were used to separate the experimental soil from the local soil. Based on the previous geographic information system for global medicinal plants (GMPGIS) technology of the research group [59], the annual average temperature during the growing period in the planting area at point A was approximately 21.1 °C. The average annual temperature was 13.6 °C, with a seasonal temperature variation of 9.919%. The annual average relative humidity was 5.970%, average annual sunshine duration was 157.49 W/m^2^, and average annual precipitation was approximately 405 mm.

### 4.3. Plant Materials

In this study, the SMB strain DZ-16-105 (D105) was used for asexual propagation. In December 2021, roots of D105 with a diameter > 6 mm were selected, cut into 6–9 cm pieces, and placed in a foam box filled with nutritious soil (Shouguang Jiaheng Agricultural Technology Co., Ltd., Linyi, China) for seedling cultivation. In March 2022, the root segments of D105 were transplanted into ridges filled with soil from the five sites. Fifteen plants were cultivated in two lines at a spacing of 30 cm in each ridge. In February 2023, roots were harvested and dried to a constant weight at 60 °C.

### 4.4. Soil Sampling and Elemental Analysis

Before cultivation, five soil samples were collected from each subsoil layer using the quartering method (Figure 1c). Impurities were removed from the soil samples; they were then air-dried, crushed, ground, sieved through a 2 mm sieve, and collected in Ziploc bags until required for further analysis. Soil elemental analysis was conducted on the ground soil using the standard method described by Bao Shidan [60].

### 4.5. Extraction and UHPLC-QTOF-MS Analysis

Salvianolic acid B and tanshinone IIA (purchased from the Chinese Institute for Food and Drug Control) were weighed, and 100% methanol was added to prepare standard concentrations of 19.193, 28.7895, 57.579, 115.158, and 230.316 mg/mL, and 41.67, 62.5, 125.0, 250.0, and 500.0 μg/mL, respectively. Phenolic acid and tanshinone were simultaneously separated using HPLC, and quantitative analysis was performed based on the standard curves of salvianolic acid B and tanshinone IIA (Figure S3). The total tanshinone content of SMBs was calculated using tanshinone IIA as a reference and multiplied by a correction factor of 3.49. After weighing the SMB powder (0.5 g), 10 mL of 100% methanol was placed in a 25 mL centrifuge tube (wrapped in tin foil and treated in the dark), and SMB extract was obtained by ultrasonic treatment for 30 min. The supernatant was sucked through a 0.25 um microporous filter membrane, placed in a 2 mL brown liquid vial, and stored in the dark at 4 °C for later use. Five SMB samples per soil type were analyzed (n = 15), and chemical fingerprints were constructed. High-performance liquid chromatography–electrospray ionization hybrid linear ion trap mass spectrometry (HPLC-ESI LQT Orbitrap/MS) was used for chromatographic peak identification and mass fragment characterization of the effective constituents of SMB. The chromatographic conditions were as follows: Diamonsil C18 column (4.6 mm × 150 mm, 3.5 μm); mobile phase: 0.1% aqueous phosphoric acid solution (A) and acetonitrile A (B); gradient elution: 0–8 min: 14–19% B, 8~14 min: 19% B, 14~34 min: 19~21% B, 34~35 min: 21~90% B, 35~40 min: 90% B; flow rate: 1.0 mL·min^−1^; column temperature: 26 °C, detection wavelength: 286 nm; injection volume: 2 μL.

### 4.6. Statistical Analysis

The test data were preprocessed using Excel 2019 (Microsoft Corporation, Redmond, WA, USA), and the results were expressed as mean ± standard deviation (mean ± SD). A one-way analysis of variance (one-way ANOVA) was used for the statistical analysis. The least significant difference (LSD) test was used for homogeneous variance, and Dunnett’s *t*-test was used for heterogeneous variance. *p* < 0.05 and *p* < 0.01 were considered statistically significant. The area’s largest ecological similarity in Shandong of the SMB was drawn using ArcGIS 10.8 (Esri, Redlands, CA, USA). A heat map was drawn using TBtools II v2.034 rendering. Interactive Mantel test correlation heat maps were constructed using an online tool (https://www.chiplot.online (accessed on 7 June 2024)). SPSS 25.0 and Origin 2022 were used for statistical analysis and figure generation.

## 5. Conclusions

Using HPLC-MS, ten water- and twelve lipid-soluble chemical components in SMB were identified. The specificity of the soils in the five main production areas led to the differential accumulation of lipid- and water-soluble components in the SMB. Correlation analysis indicated that soil elements and heavy metal pollutants had a certain tendency toward the biosynthesis of lipid and water-soluble components of SMB. Among them, AK was positively correlated with water-soluble components of SMB roots but negatively correlated with most lipid-soluble components. Interestingly, soil heavy metal pollutants As, Cd, and Cu were significantly negatively correlated with the water-soluble components of SMB roots but were significantly positively correlated with most lipid-soluble components. This suggests that these pollutants might influence the balance between the two types of compounds. This study illustrates the effects of differences in soil properties on the accumulation of the active components of SMB. These results provide data support for improving the biosynthesis and accumulation of lipid- or water-soluble components in SMB roots after directed formula fertilization in SMB-cultivated soil.

## Figures and Tables

**Table 1:** Quantitative comparative analysis of salvianolic acid B and total tanshinone of SMB roots harvested from different soils.

Soil Site	Salvianolic Acid B (%)	Total Tanshinone (%)	Salvianolic Acid B: Total Tanshinone
A	7.06 ± 0.16 d	1.00 ± 0.01 a	7.03
B	7.11 ± 0.18 d	0.76 ± 0.03 b	9.34
C	8.43 ± 0.04 c	0.95 ± 0.04 a	8.91
D	9.15 ± 0.07 b	1.03 ± 0.03 a	8.87
E	9.69 ± 0.19 a	0.61 ± 0.01 c	15.99

Total tanshinone was the sum of cryptotanshinone, tanshinone I, and tanshinone IIA. Significant differences between soils were indicated by the least significant difference (LSD) test, with different lowercase letters indicating *p* < 0.05.

**Table 2:** Identification of compounds in SMB by UHPLC-QTOF-MS.

Number	Patient ID	t_R_ (min)	Mass Ion (*m/z*)	Data-Dependent MS Data (*m*/*z*)	Identidication	Cite
1	Sm4	3.49	[M − H]^−^ 197.05	174.96, 146.97	Danshensu	[24,30,31]
2	Sm7	7.00	[M − H]^−^ 179.03	164.93, 135.04, 104.96	Caffeic acid	[30,31]
3	Sm9	9.86	[M + Na]^+^ 337.10	302.97, 286.99, 247.00, 231.05	Salvianolic acid F	[30,31]
4	Sm10	11.56	[M − H]^−^ 735.16	735.16, 294.89, 174.96, 146.97	Hydrosalvianolic acid B	[30]
5	Sm15	12.70	[M − H]^−^ 359.08	197.04, 161.02	Rosmarinic acid	[24,30,31,32,33,34]
6	Sm16	13.06	[M − H]^−^ 537.11	493.12, 294.89, 174.96, 146.97	Lithospermic acid	[24,31,34]
7^a^	Sm18	13.81	[M − H]^−^ 717.16	635.08, 294.89, 174.96, 146.97	Salvianolic acid B	[30,31,32,33]
8	Sm19	14.23	[M + Na]^+^ 741.15	425.09, 205.06	Salvianolic acid E	[24]
9	Sm20	14.8	[M − H]^−^ 717.15	551.13, 362.88, 226.92, 174.96	Iso salvianolic acid B	[30]
10	Sm24	15.61	[M − H]^−^ 551.12	424.85, 248.90, 226.92, 180.91	Methyl salvianolic acid I/H	[30]
11	Sm27	16.54	[M + Na]^+^ 319.10	301.08, 279.10, 261.06,173.01	Tanshinone VI	[30]
12	Sm31	18.21	[M + H]^+^ 274.27	212.12, 173.01, 125.99, 110.01	Tanshinone IIB	[30,31,35]
13	Sm33	19.50	[M + H]^+^ 341.14	212.12, 173.01, 143.00, 125.99	Methyldihydronortanshinonate	[30]
14	Sm40	20.37	[2M + H]^+^ 619.31	441.45, 321.15, 205.06	Tanshinaldehyde	[30,31]
15	Sm43	21.19	[M + H]^+^ 279.10	212.12, 173.01, 143.00, 125.99	15,16-Dihydrotanshinone I	[30]
16	Sm46	22.01	[M + H]^+^ 281.11	173.01, 125.99	Trijuganone B	[30,31,35]
17	Sm47	22.50	[M + H]^+^ 339.12	212.12, 173.01, 143.00, 125.99	Methyl tanshinonate	[30]
18	Sm48	24.16	[M + H]^+^ 297.15	279.00, 251.00	Cryptotanshinone	[29,30,33]
19	Sm49	25.22	[M + H]^+^ 279.10	212.12, 173.01, 143.00, 125.99	1,2-Dihydrotanshinone I	[30]
20	Sm50	25.36	[M + H]^+^ 279.10	212.12, 173.01, 143.00, 125.99	3,4-dihydrotanshinone I	[30]
21 ^a^	Sm53	27.24	[M + H]^+^ 295.15	125.99	Tanshinone IIA	[24,29,30,31,32,33,35]
22	Sm54	28.11	[M + H]^+^ 283.17	212.12, 202.09, 125.99, 110.01	Miltirone	[31,35]

^a^ Positively identified via comparison with reference standards.

## Data Availability

Data from this study are included in this article/Supplementary Materials; further inquiries can be directed to the corresponding author.

## Supplementary Materials

The following supporting information can be downloaded at: https://www.mdpi.com/article/10.3390/molecules29174016/s1. Figure S1. UV chromatogram of each component at 270 nm; Figure S2. UV chromatogram of each component at 286 nm; Figure S3: Salvianolic acid B and tanshinone IIA standard curves and chemical formulas. (a,b) Standard curves of salvianolic acid B and tanshinone IIA, the limits of salvianolic acid B were 1.352–17.291%, and the limits of tanshinone IIA were 0.038–0.484%; Table S1: Clustering analysis of 56 chemical components of SMBs from five soils; Table S2: Qualitative identification of 22 chemical compounds in SMB based on UPLC-QTOF; Table S3: DCA table of origin content-active ingredient data.
