# Supplementary material for: UHPLC-QTOF-MS-Based Targeted Metabolomics Provides Novel Insights into the Accumulative Mechanism of Soil Types on the Bioactive Components of Salvia miltiorrhiza
Source: Molecules. 2024 Aug 25;29(17):4016. doi: 10.3390/molecules29174016 (PMC11396046; doi:10.3390/molecules29174016)
Supplement: Supplementary file 1 [file molecules-29-04016-s001.zip › molecules-3149325-supplementary.pdf]

Supplementary Material

UHPLC-QTOF-MS-based targeted metabolomics provides novel insights into accumulative mechanism of soil types on the bioactive components of *Salvia miltiorrhiza*

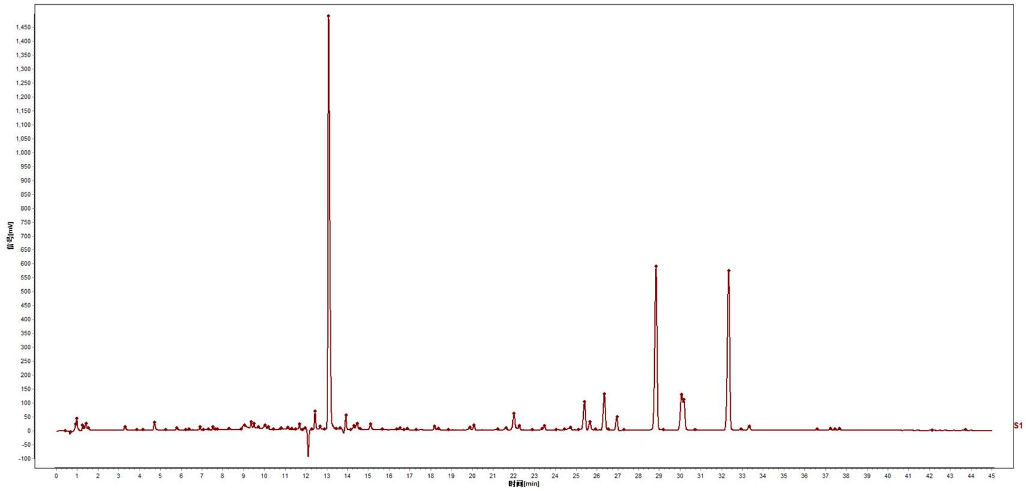

Figure S1. UV chromatogram of each component at 270nm

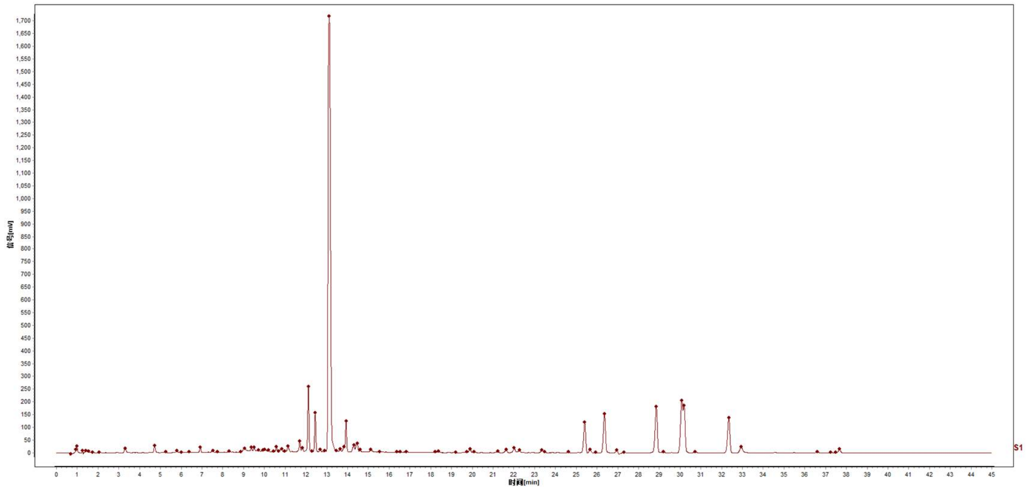

Figure S2.. UV chromatogram of each component at 286nm.

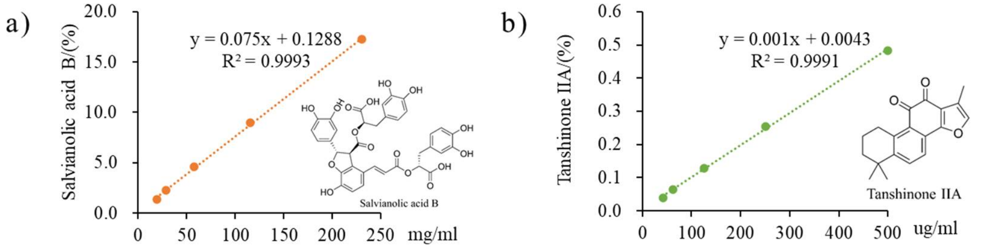

Figure S3. Salvianolic acid B and Tanshinone IIA standard curves and chemical formulas. **a)-b)** Standard curves of Salvianolic acid B and Tanshinone IIA, the limits of Salvianolic acid B were 1.352%-17.291%, and the limits of Tanshinone IIA were 0.038%-0.484%.

Table S1. Clustering analysis of 56 chemical components of SMBs from 5 soils

Patient ID	A1	A2	A3	B1	B2	B3	C1	C2	C3	D1	D2	D3	E1	E2	E3
Sm1	1.310	1.050	0.301	1.543	1.307	0.856	−0.785	−1.535	−1.401	−0.378	−0.360	−0.315	−0.395	−0.804	−0.394
Sm2	−0.492	−0.804	0.435	0.288	0.426	−0.552	−1.490	−1.152	−1.231	−0.083	0.066	−0.081	1.615	1.479	1.575
Sm3	0.893	0.733	−0.179	1.644	1.589	0.648	−1.089	−1.651	−1.659	−0.024	0.025	0.030	−0.274	−0.417	−0.268
Sm4	−0.033	−0.143	−0.382	−0.228	−0.206	−0.168	−1.377	−1.490	−1.606	0.382	0.437	0.470	1.139	1.498	1.707
Sm5	−0.567	−0.768	0.132	−1.610	−1.582	−0.581	1.246	0.966	0.879	−0.510	−0.422	−0.501	1.211	0.984	1.123
Sm6	0.760	0.613	0.925	−0.074	−0.178	−1.019	0.145	−0.766	−0.379	−1.356	−1.238	−1.359	1.221	1.336	1.368
Sm7	−0.028	−0.177	−0.212	−0.473	−0.485	−0.089	−1.371	−1.399	−1.385	0.254	0.329	0.317	1.608	1.514	1.597
Sm8	−0.728	−0.750	−0.409	−0.924	−0.512	−0.384	1.915	1.854	1.779	−0.625	−0.079	−0.080	−0.785	−0.412	0.138
Sm9	−0.637	−0.650	−0.536	−0.768	−0.739	−0.622	0.403	0.338	0.274	−1.024	−0.505	−0.483	1.220	1.143	2.584
Sm10	−0.046	−0.156	−0.891	−0.129	0.706	0.061	−1.604	−1.648	−1.610	0.720	0.805	0.918	0.936	0.882	1.056
Sm11	0.019	−0.275	0.620	−0.204	−0.149	−1.181	0.259	0.578	0.683	−1.399	−1.378	−1.472	1.561	1.081	1.257
Sm12	−0.742	−0.866	−0.110	−1.554	−1.116	−1.432	1.471	1.553	0.940	0.534	0.781	0.672	0.154	−0.372	0.088
Sm13	−0.439	−0.518	−0.320	−0.446	−0.327	−0.423	−1.955	0.705	−1.665	0.494	0.493	0.487	1.422	1.157	1.333
Sm14	−0.993	−1.108	−1.014	−1.108	−0.946	−0.894	−0.137	0.307	0.149	0.327	0.395	0.353	1.676	1.380	1.613
Sm15	−0.850	−0.908	−0.616	−0.905	−0.865	−0.860	0.409	0.448	0.392	−0.208	−0.171	−0.174	0.034	2.081	2.193
Sm16	−0.813	−0.809	−0.621	−0.824	−0.818	−0.806	1.035	1.090	1.052	−0.406	−0.394	−0.390	−0.736	1.689	1.751
Sm17	0.808	0.466	0.912	−0.304	−0.548	−0.643	0.031	0.187	0.015	−1.279	−1.345	−1.457	−0.255	1.696	1.716
Sm18	−1.200	−1.295	−0.816	−1.296	−1.126	−0.727	0.053	0.219	0.130	0.633	0.821	0.809	0.982	1.294	1.518
Sm19	−0.509	−0.610	−0.511	−0.848	−0.851	−0.755	0.110	0.111	0.172	−0.618	−0.587	−0.575	1.825	1.757	1.888
Sm20	−0.098	−0.332	−0.376	−0.229	−0.235	0.203	−1.501	−1.462	−1.595	0.404	0.519	0.467	1.488	1.277	1.469
Sm21	−0.096	−0.710	2.751	−0.417	−1.138	−0.060	−1.784	−0.385	−0.197	0.147	0.126	0.107	0.795	0.532	0.330
Sm22	−0.086	−0.252	−0.148	−0.668	−1.269	−0.867	−0.719	−1.177	−1.381	0.885	1.060	0.988	1.169	1.249	1.217
Sm23	0.717	0.615	−0.012	0.264	0.603	0.941	−0.179	−0.066	−0.626	0.930	0.808	1.089	−1.537	−1.786	−1.762
Sm24	0.554	0.433	0.073	−0.662	−0.456	−0.376	0.281	0.521	0.496	1.269	1.165	1.370	−1.555	−1.603	−1.511
Sm25	−0.279	−0.376	0.540	−0.504	−0.555	−0.470	1.260	1.855	2.302	−0.642	−0.652	−0.624	−0.640	−0.637	−0.579
Sm26	0.844	0.709	0.384	−0.607	−0.748	−0.627	0.448	0.779	0.876	0.912	0.691	1.035	−1.626	−1.594	−1.475
Sm27	0.154	0.082	0.826	1.135	1.054	1.837	−1.243	0.493	0.482	−1.322	−1.327	−1.315	−0.317	−0.351	−0.187
Sm28	0.584	1.558	0.307	−0.294	−0.358	−0.038	−0.906	0.049	0.663	1.328	1.273	0.162	−1.475	−1.475	−1.377
Sm29	0.397	−0.086	0.388	−0.050	−0.126	0.135	0.076	0.661	0.730	1.074	0.947	1.138	−1.777	−1.853	−1.654
Sm30	0.754	0.586	−0.164	−0.419	−0.457	−0.524	0.200	0.124	0.161	1.416	1.287	1.459	−1.538	−1.510	−1.373

Sm31	0.991	1.059	0.616	−0.084	−0.255	0.010	0.281	0.151	0.285	0.763	0.523	0.977	−1.810	−1.849	−1.659
Sm32	0.049	−0.084	2.253	0.759	0.783	1.050	−1.873	0.350	0.372	−0.921	−0.936	−0.933	−0.316	−0.341	−0.211
Sm33	0.940	0.802	0.391	−0.860	−1.047	−0.734	0.305	0.420	0.526	1.136	0.896	1.301	−1.449	−1.487	−1.139
Sm34	0.641	0.412	1.393	−0.095	−0.230	0.443	−1.474	1.500	1.542	−0.132	−0.192	−0.252	−1.265	−1.334	−0.959
Sm35	0.760	0.655	0.249	−0.450	−0.457	−0.135	0.763	0.793	0.791	0.878	0.841	0.477	−1.508	−1.855	−1.801
Sm36	0.025	−0.037	1.451	1.120	0.785	1.921	−1.564	0.335	0.499	−0.758	−0.756	−0.786	−0.699	−0.985	−0.549
Sm37	0.871	0.673	1.154	0.636	0.525	1.517	−1.540	0.637	0.630	−0.450	−0.469	−0.441	−1.279	−1.330	−1.132
Sm38	0.517	0.399	0.268	−0.677	−0.728	−0.446	0.678	0.385	0.406	1.253	1.196	1.290	−1.569	−1.539	−1.431
Sm39	0.793	0.707	0.350	−0.878	−0.932	−0.753	0.450	0.474	0.524	1.154	1.057	1.242	−1.432	−1.428	−1.328
Sm40	0.637	0.530	0.141	−0.831	−0.822	−0.640	0.175	0.601	0.606	1.277	1.260	1.276	−1.423	−1.427	−1.360
Sm41	1.294	1.009	0.483	0.162	−0.079	0.894	−0.978	−0.051	−0.006	0.702	0.615	0.777	−1.682	−1.728	−1.413
Sm42	1.045	0.884	0.802	0.853	0.616	−0.066	−0.464	0.571	0.650	0.131	0.004	0.250	−1.845	−1.861	−1.569
Sm43	1.003	0.879	1.751	0.346	0.186	1.042	−1.524	0.616	0.714	−0.555	−0.660	−0.455	−1.177	−1.191	−0.974
Sm44	0.934	0.850	0.673	−0.606	−1.083	−0.372	0.326	0.137	0.407	1.047	0.566	1.471	−1.526	−1.629	−1.193
Sm45	−0.067	−0.313	−1.085	1.421	1.365	1.637	−0.448	−0.242	−0.145	0.512	0.570	0.622	−1.299	−1.373	−1.155
Sm46	1.051	0.839	1.234	−0.263	−0.331	0.386	−0.562	1.081	1.088	0.130	0.109	0.021	−1.657	−1.644	−1.482
Sm47	0.781	0.916	0.035	−0.691	−1.077	−0.764	0.388	−0.032	0.088	1.352	0.999	1.666	−1.266	−1.419	−0.976
Sm48	1.329	1.161	0.764	−0.574	−0.627	−0.226	0.389	0.277	0.329	0.672	0.589	0.757	−1.662	−1.657	−1.521
Sm49	1.025	0.940	1.144	−1.228	−1.238	−1.116	0.256	0.467	1.826	0.169	0.121	0.238	−1.295	−0.985	−0.324
Sm50	0.712	0.512	0.205	−1.032	−1.124	−1.013	1.338	0.461	0.533	1.018	0.916	1.112	−1.274	−1.284	−1.079
Sm51	0.653	0.434	0.187	−1.169	−1.273	−1.183	1.954	0.394	0.508	0.784	0.652	0.940	−1.039	−1.054	−0.787
Sm52	0.879	0.760	0.631	−0.888	−0.969	−0.863	1.110	0.704	0.741	0.709	0.699	0.724	−1.428	−1.433	−1.375
Sm53	0.897	0.780	0.631	−0.530	−0.926	−0.423	0.920	0.156	0.312	0.940	0.577	1.265	−1.571	−1.650	−1.378
Sm54	0.778	0.703	0.332	−0.914	−0.881	−0.672	0.560	0.522	0.570	1.111	1.105	1.090	−1.451	−1.451	−1.401
Sm55	0.551	0.317	−0.467	−0.345	−0.568	−0.354	−0.059	−0.472	−0.437	1.693	1.680	1.755	−1.118	−1.136	−1.042
Sm56	0.419	0.312	−0.170	−0.987	−1.072	−0.817	0.388	0.546	0.553	1.494	1.479	1.315	−1.212	−1.174	−1.076

Table S2. Qualitative identification of 22 chemical compounds in SMBs based on UPLC-QTOF

Number	Cpd.	Patient ID	A1	A2	A3	B1	B2	B3	C1	C2	C3	D1	D2	D3	E1	E2	E3
1	Danshensu	Sm4	41.4	40.1	37.3	39.1	39.4	39.8	25.5	24.2	22.8	46.3	47.0	47.4	55.3	59.5	62.0
2	Caffeic acid	Sm7	34.5	33.2	32.9	30.6	30.4	34.0	22.5	22.3	22.4	37.0	37.7	37.6	49.1	48.3	49.0
3	Salvianolic acid F	Sm9	31.7	31.3	35.4	27.0	28.0	32.3	69.8	67.4	65.1	17.6	36.6	37.4	99.7	96.8	149.5
4	Hydrosalvianolic acid B	Sm10	49.4	47.7	35.9	48.1	61.4	51.1	24.5	23.8	24.4	61.6	63.0	64.8	65.1	64.2	67.0
5	Rosmarinic acid	Sm15	923.3	891.3	1053.5	892.9	915.2	918.0	1622.2	1643.7	1612.9	1279.5	1300.3	1298.8	1413.7	2549.4	2612.0
6	Lithospermic acid	Sm16	24.2	25.7	85.3	20.8	22.7	26.7	610.0	627.5	615.6	153.4	157.1	158.4	48.8	817.5	837.0
7	Salvianolic acid B	Sm18	9022.1	8889.1	9560.8	8887.5	9126.9	9686.4	10779.8	11013.3	10888.4	11594.4	11858.2	11841.1	12084.0	12520.7	12835.2
8	Salvianolic acid E	Sm19	28.2	26.7	28.2	23.1	23.0	24.5	37.5	37.5	38.5	26.6	27.0	27.2	63.4	62.4	64.3
9	Iso salvianolic acid B	Sm20	246.7	236.8	235.0	241.2	240.9	259.4	187.5	189.1	183.5	267.9	272.8	270.6	313.7	304.8	312.9
10	Methyl salvianolic acid I/H	Sm24	302.4	293.3	266.0	210.5	226.1	232.1	281.8	299.9	298.0	356.4	348.6	364.1	143.0	139.4	146.3
11	Tanshinone VI	Sm27	146.8	142.9	182.7	199.1	194.8	236.6	72.2	164.9	164.3	67.9	67.7	68.3	121.6	119.8	128.5
12	Tanshinone IIB	Sm31	300.5	303.8	282.2	248.0	239.6	252.6	265.8	259.5	266.0	289.4	277.7	299.8	163.6	161.7	171.0
13	Methyl Methyldihydronortanshinonate	Sm33	948.1	928.1	868.9	688.7	661.7	706.8	856.5	873.1	888.4	976.3	941.8	1000.0	603.7	598.3	648.4
14	Tanshinaldehyde	Sm40	354.4	342.9	301.2	197.1	198.0	217.5	304.8	350.4	351.0	422.9	421.1	422.8	133.6	133.2	140.4
15	15,16-Dihydrotanshinone I	Sm43	1565.6	1530.8	1775.9	1381.1	1336.0	1576.8	855.1	1456.9	1484.4	1127.8	1098.2	1155.9	952.7	948.9	1009.8
16	Trijuganone B	Sm46	3500.9	3374.9	3609.4	2720.8	2681.0	3106.2	2543.7	3518.9	3522.8	2954.6	2942.1	2889.6	1893.7	1901.5	1997.5
17	Methyl tanshinonate	Sm47	687.4	698.5	626.1	566.4	534.7	560.5	655.2	620.6	630.5	734.4	705.3	760.1	519.2	506.6	543.0
18	Cryptotanshinone	Sm48	14075.6	13656.6	12670.8	9341.3	9208.6	10207.5	11738.2	11458.8	11588.4	12441.7	12233.6	12651.4	6633.8	6647.6	6985.9
19	1,2-Dihydrotanshinone I	Sm49	105.7	104.9	106.9	83.1	83.0	84.2	98.0	100.1	113.8	97.1	96.6	97.8	82.4	85.5	92.2
20	3,4-dihydrotanshinone I	Sm50	2369.0	2286.1	2158.5	1644.9	1607.0	1653.1	2629.0	2264.7	2294.6	2496.0	2453.6	2535.3	1544.4	1540.4	1625.3
21	Tanshinone IIA	Sm53	11051.6	10840.5	10571.6	8481.5	7766.9	8674.2	11093.2	9716.7	9998.2	11129.3	10474.6	11714.6	6604.6	6462.4	6953.3
22	Miltirone	Sm54	770.7	753.6	669.0	384.5	391.9	439.7	720.8	712.2	723.1	846.7	845.3	841.9	261.8	262.0	273.4

Table S3. DCA analysis table of origin content-active ingredient data

DCA	Eigenvalues	Decorana values	Axis lengths
DCA1	0.0259	0.0268	0.9888
DCA2	0.0036	0.003	0.5925
DCA3	0.0065	0.0003	0.4372
DCA4	0.0066	0.0001	0.6388
